# Aqueous Extract of *Ficus bengalensis* Linn. Bark for Inflammatory Bowel Disease

**DOI:** 10.4103/0975-1483.63149

**Published:** 2010

**Authors:** MA Patel, PK Patel, MB Patel

**Affiliations:** *Department of Pharmacology, C. K. Pithawala Institute of Pharmaceutical Sciences and Research, Surat-395 007, Gujarat, India*; 1 *Department of Pharmacology, Shri Sarvajanik College of Pharmacy, Mahesana, Gujarat, India*

**Keywords:** Aqueous extract, bark, *Ficus bengalensis* (Moraceae), inflammatory bowel disease

## Abstract

The present study was designed to evaluate the effects of aqueous extract of *Ficus bengalensis* Linn. bark (AEFB) on inflammatory bowel disease (IBD). Effects of AEFB were studied on 2, 4, 6-trinitrobenzenesulfonic acid (TNBS, 0.25 ml 120 mg/ml in 50% ethanol intrarectally, on first day only)-induced IBD in rats. Effects of co-administration of prednisolone (2 mg/kg) and AEFB (250, 500 mg/kg) for 21 days were also evaluated. Various physical parameters including body weight, food, and water intake measured on 1st and 21st days. At end of the experiment, various histopathological indexes are assessed. The colon homogenate malondialdehyde (MDA), myeloperoxidase (MPO), superoxide dismutase (SOD), and nitric oxide (NO) levels and % mast cell protection in mesentery were also measured. In our study, we found that AEFB has a significant protective effect in the inflammatory bowel disease as compared to prednisolone in rats.

## INTRODUCTION

Inflammatory bowel disease (IBD) encompasses many chronic, relapsing inflammatory disorders involving the gastrointestinal tract.[1] There are two primary types of IBD, namely Crohn’s disease and ulcerative colitis. In IBD, the intestine (bowel) becomes inflamed, often causing recurring abdominal cramps, and diarrhea. Among the pathological findings associated with IBD are increases in certain inflammatory mediators, signs of oxidative stress, a deranged colonic milieu, abnormal glycosaminoglycan content of the mucosa, increased intestinal permeability, increased sulfide production, and decreased methylation.[2] The available treatment choices have major limits owing to associated adverse effects and compliance issues.[1] As a result, there is high prevalence of complementary and alternative medicines for treating the mentioned disease.

*Ficus bengalensis* Linn. (Family: Moraceae) is a reputed plant in ayurvedic medicine and commonly known as ‘banayan tree’ in ayurvedic literature. Milky juice from stem, seed, or fruits of the plant is applied externally in rheumatism and to the soles of feet when inflamed, internally used in dysentery and diarrhea.[3] All the parts of the plant have astringent, anti-inflammatory, antiarthritis, and antidiarrheal activities. The bark is also used in diarrhea and dysentery. Latex is useful in hemorrhage, diarrhea, and dysentery, as well as in hemorrhoid and inflammation.[4] Severe inflammation and diarrhea are the characteristics of IBD. So, with the light of the folkloric usage of the bark mainly in inflammation and diarrhea, this study carried out to evaluate the efficacy of the aqueous extract of *F. bengalensis* bark (AEFB) in IBD.

## MATERIALS AND METHODS

### Plant materials

The stem bark of *F. bengalensis* Linn. was collected from south Gujarat region. The plant was identified and authenticated by Department of Bioscience, VNSGU, Gujarat, India (Voucher specimen number is HMG/0404/2007).

### Preparation of plant extract

The stem bark of the plant was air dried, reduced to coarse powder, macerated with distilled water for 48 h, filtered and the filtrate was evaporated under reduce pressure to obtain dry extract (aqueous extractive value 4.8% w/w). The extract was stored in cool and dry place and was used for pharmacological evaluation. The extract was suspended in 0.5% carboxyl methylcellulose (CMC) prior to administration. Various phytoconstituents qualitatively were determined in aqueous extract according to Wagner and Bladt, 1996.[5]

### Animals

Adult albino (Wistar strain) rats of either sex weighing between 200 and 250 g housed in standard conditions of temperature (22 ± 2°C), relative humidity (55 ± 5%), and light (12 h light/dark cycles) were used. They were fed with standard pellet diet and water *ad libitum*. Animals were approved by the Institutional Animal Ethics Committee (IAEC) according to the regulation of Committee for the Purpose of Control and Supervision of Experiments on Animals (CPCSEA). Throughout the experiments, animals were handled according to the suggested ethical guideline for the care of laboratory animals.

### Chemicals

TNBS (2, 4,6 -trinitrobenzenesulfonic acid), prednisolone, compound 48/80 were bought from the Sigma-Aldrich Chemical Co., USA and all other chemicals were purchased from the Himedia Pvt. Ltd, India. Biochemicals kits were purchased from the Span Diagnostic Ltd, Surat, India.

### Acute toxicity testing

Albino rats of either sex weighing 230-240 g selected by the random sampling technique were used in the study. Acute oral toxicity study was performed as per Organisation for Economic Co-operation and Development (OECD)---423 guideline. The animals were fasted overnight, provided only water, after which the aqueous extract of *F. benglensis* (AEFB) was administered to respective groups orally at the dose level of 5 mg/kg body weight by gastric intubation and the groups were observed for 14 days. If mortality was observed in two or three animals, then the dose administered was assigned as a toxic dose. If mortality was observed in one animal, then the same dose was repeated again to confirm the toxic dose. If mortality was not observed, the procedure was repeated for further higher dose such as 50, 300, and 2000 mg/kg body weight. The animals were observed for toxic symptoms such as behavior changes, locomotion, convulsion, and mortality for 72 h.

### Experimental protocol

The study comprised five different groups of six animals each as follows:[6]

- Control: Saline treated;
- Model: TNBS (2, 4,6 -trinitrobenzenesulfonic acid, 0.25 ml, 120 mg/ml in 50% ethanol, intrarectally) on first day only;
- Prednisolone: TNBS (0.25 ml, 120 mg/ml in 50% ethanol, intrarectally), on 1st day only and prednisolone (2 mg/kg, p.o.) treatment continued till 21st day;
- AEFB (250 mg/kg): TNBS (0.25 ml, 120 mg/ml in 50% ethanol, intrarectally) on 1st day only + AEFB (250 mg/kg, p.o.) treatment continued till 21st day;
- AEFB (500 mg/kg): TNBS (0.25 ml, 120 mg/ml in 50% ethanol, intrarectally) on 1st day only + AEFB (500 mg/kg, p.o.) treatment continued till 21^st^day.

TNBS was delivered by a Teflon cannula (outside diameter 1.2 mm, inserted 8 cm) through the anus of each rat. Ethanol evokes an acute inflammatory response that resolves spontaneously after 1 week. Therefore, we preferred to include a saline-treated group as a negative control instead of an ethanol-treated group. Various physical parameters like body weight, food intake, and water intake were measured on 1^st^ and 21^st^ days. On 21^st^ day, animals were sacrificed by cervical dislocation and dissected open to remove GIT (from stomach to anus). GIT was flushed gently with saline and cut open. Weight of the colon taken was measured and then the colon mucosa damage index (CMDI) and the histopathological score i.e. disease activity index (DAI) were evaluated. Colon samples were taken for determinations of myeloperoxidase (MPO) and superoxide dismutase (SOD) activity, malondialdehyde (MDA) and nitric oxide (NO) content level. The percentage protection of the mast cell degranulation in the mesentery of intestine of the rat was also measured.

### Assessment of the colon mucosa damage index (CMDI)

The colon segment taken 10 cm proximal to anus of the sacrificed rats was excised longitudinally, was rinsed with saline buffer, and fixed on a wax block. Each colon was observed and evaluated by two independent observers. Macroscopic scoring was done evaluated according to the formula of CMDI reported by Wei *et al*. 2003.[7] Briefly describe as follows: (1) 0-normal mucosa, 1-mild hyperemia, no erosion or ulcer on the mucosal surface, (2) 2-moderate hyperemia, erosion appearing on the mucosal surface, (3) 3-severe hyperemia, necrosis, and ulcer on the mucosal surface with the major ulcerative area extending < 40%, (4) 4-severe hyperemia, necrosis, and ulcer on the mucosal surface with the major ulcerative area extending >40%.

### Assessment of disease activity index (DAI)

The colon tissue samples taken for histology were fixed overnight in 4% neutral buffered formalin, processed, sectioned (4 µm thick), and stained with hematoxylin and eosin. Each colon sample was observed and evaluated by two independent observers. To assess, the histopathological score was assessed according to the modified model of the system reference given by Wei *et al*. 2003 which are as follows: (1) the infiltration of acute inflammatory cells: 0-no, 1-mild increasing, 2-severe increasing; (2) the infiltration of chronic inflammatory cells: 0-no, 1-mild increasing, 2-severe increasing; (3) the deposition of fibrotin protein: 0-negative, 1-positive; (4) the submucosa edema: 0-no, 1-patchy-like, 2 fusion-like; (5) the epithelium necrosis: 0-no, 1-limiting, 2-widening; (6) the epithelium ulcer: 0-negative, 1-positive.

### Determination of the MPO, SOD activity and MDA, NO content in the colon

The colon sample was homogenized (50 gm/L) in 50 mmol/l ice-cold potassium phosphate buffer (pH 6.0) containing 0.5% of hexadecyltrimethylammonium bromide. The homogenate was first frozen and thawed thrice for three times, and then centrifuged at 4000 rpm for 20 min at 4°C for the measurement of myelopeoxidase (MPO) activity. MPO, a marker of neutrophil migration was estimated by measuring H_2_O_2_ -dependent oxidation of O dianisidine.[8]

For the determination of SOD activity and MDA, NO contents, the colon sample was homogenized in ice-cold phosphate buffer saline (pH 7.4) and centrifuged at 3000 rpm for 10 min at 4°C. The clear supernatant was used for the assay of MDA which is the indicator of lipid peroxidation,[9] and the endogenous antioxidant enzyme SOD was estimated according to Misra and Fridovich, 1972.[10]

NO was determined according to the method of Yamamoto *et al*.[11] NO is produced by Nitric oxide synthatase (NOS). NOS convert arginine to citruline, during this reaction NO is produced. NO reacts with *N*-1-napthylethylenediamine dihydrochloride and sulphanilamide to give azo compound. The color intensity of that azo compound was measured at 540 nm. The quantity was measured by standard curve and reported as nmoles/mg protein.

### % Mesenteric mast cell protection

% Mast cell protection is suggested by a decrease in the degranulation of the mast cell. Mesentery of intestine from obtained the animals was removed and placed in the Ringer Locke solution (NaCl 0.9%, KCI 0.042%, CaCl_2_ 0.024%, NaHCO_3_ 0.015%, and dextrose 0.1%). Then it was stained and fixed with the 4% formaldehyde containing 0.1% toluidine blue. %Mast cell protection was evaluated microscopically at 40 magnification.[12]

### Statistical analysis

Data obtained from the animal experiments were expressed as the mean ± SEM of six observations. The statistical difference was evaluated by one-way ANOVA. Differences were accepted as statistically significant when *P*< 0.05.

## RESULTS

The yield of AEFB was estimated to be 4.8% w/w and preliminary phytochemical analysis showed that it contained presentation of glycosides, carbohydrates, flavonoids, triterpenoids, tannins, and phenolic compounds. No mortality and the sign of toxicity observed at the dose of 5000 mg/kg.

### Changes in the physical parameters

On 1st and 21st days, changes in the physical parameters including body weight, food intake, and water intake were measured. When compared with the control group, significant reductions in all these parameters were observed with the model group. While, however, in the animals treated with prednisolone and AEFB had no significant reduction in the body weight, food intake and water intake compared to the model group [Table 1]. However, in contrast, the weight of the colon was increased significantly in the TNBS-treated group i.e. model group compared to the normal group but it significantly decreased in the prednisolone- and AEFB-treated groups compared to the model group [Figure 1].

**Table 1:** Effect of aqueous extract of *Ficus bengalensis* on water intake, food intake, and body weight in TNBS-induced inflammatory bowel disease in rats

Groups	Reduction in body weight (g)	Reduction in food intake (g/group)	Reduction in water intake (ml/group)
Control	7 ± 1	5 ± 0.8	5 ± 1.8
Model	50 ± 2.5*	50 ± 4*	65 ± 1.2*
Prednisolone	10 ± 2#	6 ± 1.1#	5 ± 2#
AEFB (250 mg/kg)	15 ± 0.9#	10 ± 1#	9 ± 1.1#
AEFB (500 mg/kg)	13 ± 1#	8 ± 0.9#	7 ± 2.1#

Each value presented as mean ± SEM (*n*=6) (one-way ANOVA).

* Compared with control group, *P* < 0.001

# Compared with model group, *P* < 0.001

**Figure 1:**
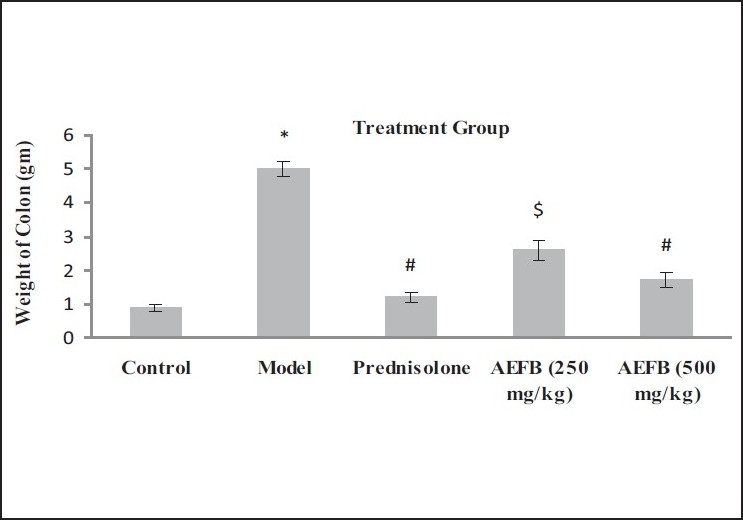
Effect of aqueous extract of *Ficus bengalensis* on the weight of the colon in TNBS-induced inflammatory bowel disease in rats. *When compared to control *P*<0.001, #When compared to model *P*<0.001, $When compared to model *P*<0.01. One-way ANOVA (n=6)

### Inflammatory changes in the mucosa of colon

The main parameters used for evaluating the degree of colonic inflammation in IBD were CMDI, DAI, and MPO activities. In this study, significant differences in CMDI, DAI, and MPO activities were found, when compared with that of normal and model groups. In prednisolone- and AEFB-treated groups CMDI and DAI- and MPO activities significantly decreased when compared with the model group [Table 2].

**Table 2:** Effects of aqueous extract of *Ficus bengalensis* on CMDI, DAI, and MPO activity in the colon tissue of TNBS-induced inflammatory bowel disease in rats

Groups	CMDI	DAI	MPO (unit/mg of protein)
Control	0.0 ± 0.0	0.7 ± 0.11	22 ± 0.9
Model	6.1 ± 0.31*	8.3 ± 0.2*	88 ±2.5*
Prednisolone	1.4 ± 0.21#	3.2 ± 0.10#	28 ±1.2#
AEFB (250 mg/kg)	2.5 ± 0.13#	4 ± 0.13#	40 ±15##
AEFB (500 mg/kg)	1.8 ± 0.18#	2.2 ± 0.11#	38 ±1.8##

(CMDI: colonic mucosal damage index, DAI: disease activity index, MPO: myeloperoxidase). Each value presented as mean ± SEM (*n*=6) (one-way ANOVA).

* When compared to control group *P*<0.001,

When compared to model group *P*<0.001.

## When compared to model group *P*<0.01.

### Oxidative changes in the colon

Severe oxidative stress induced by intrarectal administration of TNBS was shown by significant elevation of MDA and NO level and a significantly decrease in the SOD activity compared to the control group. After treatment with prednisolone and AEFB, there was a significant decrease both in MDA and NO levels and an increase in the SOD activity when compared with the model group, which showed that they have antioxidant properties [Table 3].

**Table 3:** Effect of aqueous extract of *Ficus bengalensis* on MDA and NO content, SOD activity and mast cell degranulation in TNBS-induce inflammatory bowel disease in rats

Groups	MDA (nmole/mg of protein)	NO (nmole/mg of protein)	SOD (U/min/mg of protein)	Mast cell degranulation (% protection)
Control	17 ± 0.7	12 ± 0.5	0.89 ± 0.05	80 ± 1.5
Model	88 ± 2.1^*^	28 ± 1.1^*^	0.22 ± 0.03^*^	8 ± 0.5^*^
Prednisolone	34 ± 1.4#	14 ± 0.8#	0.78 ± 0.05#	62 ± 1.0#
AEFB (250 mg/kg)	50 ± 1.7#	20 ± 0.5#	0.7 ± 0.02#	45 ± 0.8#
AEFB (500 mg/kg)	38 ± 1.8#	16 ± 0.6#	0.62 ± 0.03#	54 ± 0.9#

(MDA: malondialdehyde, NO: nitric oxide, SOD: superoxide dismutase), Each value presented as mean ± SEM (*n*=6) (one-way ANOVA). Compared with the control group *P*<0.001

# Compared with the model group *P*<0.001.

### Mesenteric mast cell degranulation

In the animals treated with the TNBS, i.e. model group, a significant increase in the mast cell degranulation occurred compared with the normal group. Treatment of the prednisolone and AEFB significantly decreased the mast cell degranulation that was significantly lower than that in of TNBS-treated animals [Table 3].

### Histopathological changes in the colon

**Figure 2:**
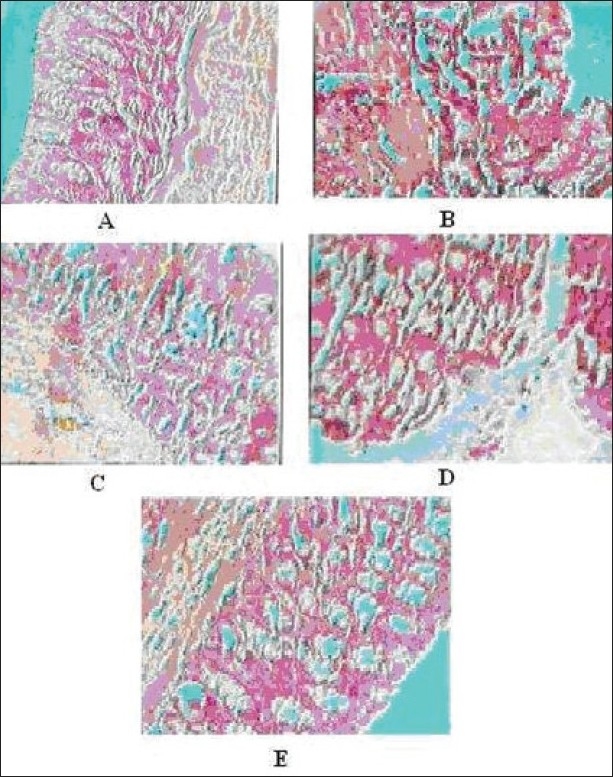
Effect of aqueous extract of *Ficus bengalensis* on tissue histopathology in TNBS-induced inflammatory bowel disease in rat. Control group: shows normal mucosa (A), model group: shows severe hyperplasia, ulcer appearing on the mucosal surface with the major ulcerative area extending >40% (B), prednisolone: shows moderate hyperplasia, no erosions appear on the mucosal surface (C), AEFB (250 mg/kg): shows mild hyperplasia and erosion appear on the mucosal surface (D), AEFB (500 mg/kg): mild hyperplasia, no ulceration on mucosa (E).

Histopathological analysis of the colon in TNBS-treated group clearly showed that there were severe hyperplasia, edema, infiltration of inflammatory cells particularly neutrophils and lymphocytes, necrosis and ulcer on the mucosal surface >40% showing the progression to severe disease [Figure 2B] compared to control group [Figure 2A]. Animals treated with the prednisolone showed mild hyperplesia and edema, infiltration of inflammatory cells was also decreased, normal mucosa, no necrosis, and no ulceration were observed [Figure 2C]. With the treatment with of AEFB, the progression of IBD were less prominent which was characterized by a decreased in hyperplasia and edema, also a declined in the infiltration of the inflammatory cells, in addition, mild necrosis, and ulceration were present in AEFB 250 mg/kg group [Figure 2D] but absent in AEFB 500 mg/kg group [Figure 2E].

## DISCUSSION

TNBS is a hapten compound, and when it is bound with a substance of high molecular tissue proteins, it will turn into an antigen. It has shown that it can elicit immunologic responses; induce generation of inflammatory bowel disease (IBD).[12,13] This model shares many of the histopathological and clinical features of human IBD and is useful for the study of the etiopathogenesis of chronic colon inflammation as well as providing an inexpensive model suitable for assessing therapeutic agents.

In IBD body weight, food intake and water intake are the important indicators of the severity of this disease. As there is a severe inflammation in the colon, the tolerability to the food and water decreases, therefore body weight is also decreased.[14] In our study we found that the treatment with AEFB decreased the weight loss by improving the tolerability to food and water intake. AEFB improved all these physical parameters that were comparable to that of prednisolone.

In IBD, weight of colon is increased due to severe inflammation and edema.[15] Animal treated with TNBS showed high colon weight compare to normal animals. Treatment with AEFB reduced colon weight of animals treated with TNBS. That shows AEFB reduced inflammation and edema.

In our study, we induced IBD by intrarectal administration of TNBS in animals. The severity of colonic inflammation in developed disease was evaluated by measuring main parameters CMDI and DAI scores and MPO activity. In present study, we found that decreased in progression of the disease pathogenesis following treatment with AEFB characterized by significantly declined in the score of CMDI and DAI compared to the model group which is also supported by the changes in the histopathology of the colon. MPO is an enzyme found in the neutrophils, and can be used as a quantitative index of inflammation in colonic tissue. MPO activity may be regarded as an index of inflammation damage. The main pathological feature of IBD is transmural infiltration of polymorphonuclear neutrophils and MPO is released from these neutrophils.[16,17] Treatment with AEFB significantly decreased the level of MPO compared to the model group which shows that AEFB decreases the infiltration of the inflammatory cells which are responsible for the increasing the progression of the disease condition.

Many studies have revealed that the increase of oxidative stress and iNOS activity was a notable feature of IBD, which resulted in a pathological cascade of free radical reactions and further yielding more oxidative free radicals. Failures of the endogenous antioxidant defense mechanisms promote formation of excessive free radicals and consequent tissue damage.[18] Parameters such as MDA, NO content, and SOD activity can be indicative of oxidative stress status of the disease. MDA level can be determining by the thiobarbituric acid reacting substance (TBARS). As observed in our study, the increase in MDA levels in the colon affected by the TNBS administration suggests enhanced lipid peroxidation that could be responsible for the tissue damage. Many studies have reported that increased iNOS activity yielded more oxidative free radicals such as peroxynitrite (ONOO^−^) to impair the structure and function of the cells.[19] Excess of NO is responsible for the increase in the disease severity by increasing vascular permeability and decrease in the antioxidant defense mechanism by inhibiting the SOD enzyme. We made an attempt to measure the NO by means of Griess assay which relies on a diazotization reaction.[11] SOD is an important endogenous antioxidant enzyme which prevents the production of free radicals.[20]

In the present study, in the animal group treated with TNBS there was observed an increase in the oxidative stress indicated by the higher MDA and NO level and as well as a decrease in the SOD activity which are responsible for the tissue damage and development of inflammation. In our study we found that for the animals treated with AEFB, both the MDA and NO levels were significantly decreased and SOD activity was significantly increased which confirmed that AEFB decreased the tissue damage and inflammation which suggested its significant antioxidant property.

Mast cell degranulation causes mucus secretion, mucosal edema, increased gut permeability, and release of various inflammatory mediators which may be responsible for some of the signs and symptoms of inflammatory bowel disease.[21] In our study a significant rise in the mast cell degranulation was observed in the TNBS-treated animals, while in AEFB-treated animals, mast cell degranulation was significantly lower. This observation clearly indicates that AEFB provides the protection against the injury produced by inflammatory mediators released from the mast cell degranulation by stabilizing it them. Prednisolone at a dose of 2 mg/kg and AEFB at doses of 250 mg/kg and 500 mg/kg also provided the protection against TNBS-induced IBD in rats. The AEFB protection by in the IBD was comparable to that of prednisolone. Therefore, it may be possible might be postulated that AEFB produced this protection by the same mechanism that of prednisolone i.e. inhibition of the infiltration of the inflammatory cells, antioxidant activity and decrease in the synthesis of the inflammatory mediators.

Various phytochemicals like phenolic compounds and flavonoids present in AEFB may be responsible for its antioxidant effect. Flavonoids also have mast cell stabilizing property.[22,23] Terpenoids[24] and flavonoids[25] have an anti-inflammatory effect, which may be responsible for the anti-inflammatory effect of AEFB in IBD. Due to the presence of flavonoids and terpenoids in the bark of *Ficus bengalensis* and the results obtained in this study might be used in to the bark of the title plant might be used for the treatment of inflammatory bowel disease.
